# Illuminating *Cannabis sativa* L.: The Power of Light in Enhancing *C. sativa* Growth and Secondary Metabolite Production

**DOI:** 10.3390/plants13192774

**Published:** 2024-10-03

**Authors:** S.M. Ahsan, Md. Injamum-Ul-Hoque, Shifa Shaffique, Akhtar Ayoobi, Md Atikur Rahman, Md. Mezanur Rahman, Hyong Woo Choi

**Affiliations:** 1 Department of Plant Medicals, Andong National University, Andong 36729, Republic of Korea; smvahsan@gmail.com (S.A.); akhtar.ayoobi@gmail.com (A.A.); 2 Department of Applied Biosciences, Kyungpook National University, Daegu 41566, Republic of Korea; injamumrassel@gmail.com (M.I.-U.-H.); shifa.2021@knu.ac.kr (S.S.); 3 ABEx Bio-Research Center, East Azampur, Dhaka 1230, Bangladesh; 4 Department of Agroforestry and Environment, Bangabandhu Sheikh Mujibur Rahman Agricultural University, Gazipur 1706, Bangladesh; 5 Institute of Genomics for Crop Abiotic Stress Tolerance, Department of Plant and Soil Science, Texas Tech University, Lubbock, TX 79409, USA; 6 Institute of Cannabis Biotechnology, Andong National University, Andong 36729, Republic of Korea

**Keywords:** cannabis, growth and development, inflorescence, light quality, light intensity, photoperiod, secondary metabolites

## Abstract

Light is crucial for higher plants, driving photosynthesis and serving as a powerful sensory signal that profoundly modulates growth, development, physiological functions, hormone activation, and biochemical pathways. Various light parameters—quality, intensity, composition, and photoperiod—exert a tremendous influence on plant growth and development, particularly in industrial hemp (*Cannabis sativa* L.). *C. sativa*, a crop of historical significance and unparalleled versatility, holds immense value in the food, fiber, and medicinal industries. The cultivation of medicinal cannabis is burgeoning in controlled environments due to evolving healthcare regulations. Optimal light conditions significantly enhance both yield and harvest quality, notably increasing the density of apical inflorescences and the ratio of inflorescence to total aboveground biomass. *C. sativa* metabolites, especially phenolic and terpene compounds and Phytocannabinoids like CBD (cannabidiol), THC (tetrahydrocannabinol), and CBG (cannabigerol), possess immense medicinal value. Secondary metabolites in *C. sativa* predominantly accumulate in the trichomes of female flowers and surrounding sugar leaves, underscoring the critical need to boost inflorescence weight and metabolite concentrations while ensuring product consistency. Different light parameters distinctly impact *C. sativa*’s metabolic profile, providing a robust foundation for understanding the optimal conditions for synthesizing specific secondary metabolites. While the effects of light measurement on various crops are well-established, scientific evidence specifically relating to light quality effects on *C. sativa* morphology and secondary metabolite accumulation remains scarce. In this review, we critically summarized how different light properties can alter cannabis growth (vegetative and reproductive), physiology and metabolism. Furthermore, the mechanisms by which specific wavelengths influence growth, development, and secondary metabolite biosynthesis in *C. sativa* are not fully elucidated, which could be a prospective task for future researchers. Our review paves the way for a profound understanding of light’s influence on *C. sativa* growth and advancements in greenhouse settings to maximize metabolite production for commercial use.

## 1. Introduction

**Figure 1:**
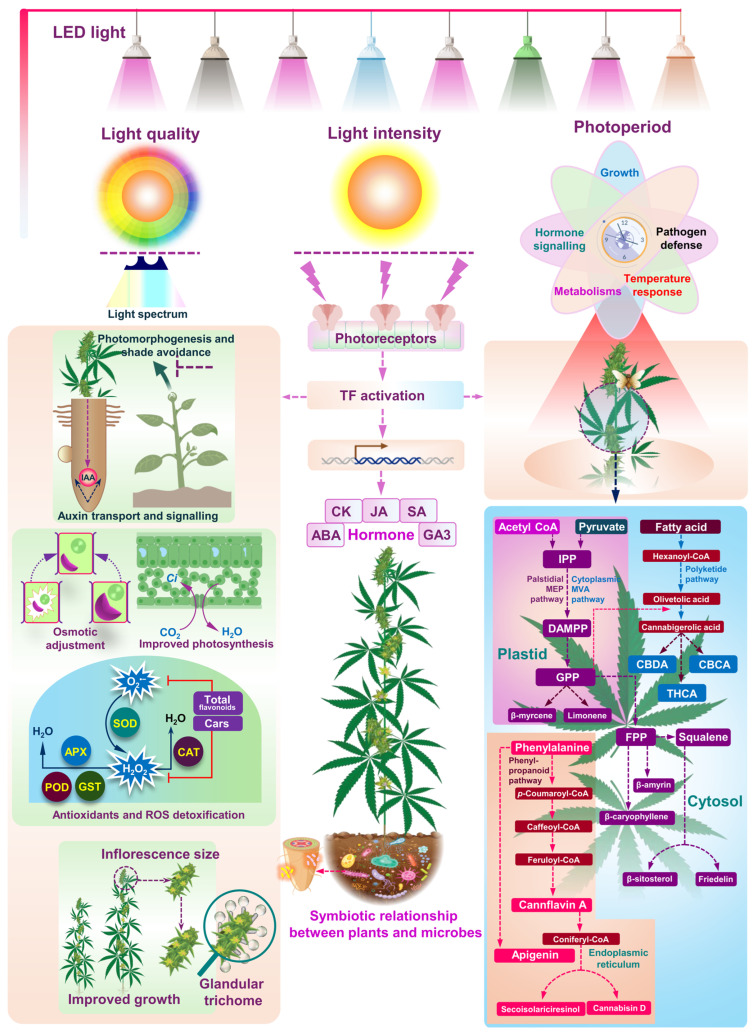
The figure illustrates the intricate effects of light quality, intensity, and photoperiod on the growth, development, and metabolic pathways of cannabis plants. Light, in terms of quality, intensity, and photoperiod, is perceived by photoreceptors, which subsequently trigger transcription factors (TFs) that regulate gene expression related to growth and development. The use of LED lights provides controlled light spectra that influence various physiological responses. Optimal wavelengths significantly impact photomorphogenesis and shade avoidance mechanisms, primarily by regulating auxin transport and signaling. The circadian clock also plays a role in coordinating growth, hormone signaling, and metabolic processes in response to light cycles. Optimal light conditions enhance photosynthesis efficiency by increasing CO_2_ and H_2_O uptake, while also promoting osmotic adjustment and strengthening the plant’s defense against reactive oxygen species (ROS) through the upregulation of detoxification enzymes such as superoxide dismutase (SOD), catalase (CAT), glutathione peroxidase (GPX), and glutathione *S*-transferase (GST). These changes, collectively contribute to vigorous plant growth, increased inflorescence size, and the development of glandular trichomes, which are important for secondary metabolite production. The plant’s hormonal responses, including auxin (IAA), cytokinin (CK), abscisic acid (ABA), gibberellin (GA3), salicylic acid (SA), and jasmonic acid (JA), are also regulated by light and play a critical role in growth and stress responses. In addition to light’s direct effects on plant physiology, the figure highlights the symbiotic relationship between cannabis plants and microbes, which can further enhance plant health and nutrient acquisition. The top-right section emphasizes the impact of different light spectra on plant responses, affecting metabolic pathways and cannabinoid production. The detailed metabolic pathways in the bottom-right panel depict the biosynthesis of cannabinoids and terpenoids. Key precursors such as acetyl CoA, pyruvate, and fatty acids enter the Palstidial MEP and cytoplasmic MVA pathways to produce isopentenyl pyrophosphate (IPP) and geranyl pyrophosphate (GPP), leading to the synthesis of primary cannabinoids like Cannabigerolic acid (CBGA). CBGA serves as a precursor for tetra-hydrocannabinolic acid (THCA), cannabidiolic acid (CBDA), and cannabichromenic acid (CBCA). The terpenoid biosynthesis pathway illustrates how intermediates like GPP and farnesyl pyrophosphate (FPP) are converted into terpenoids, including β-myrcene, limonene, β-caryophyllene, β-amyrin, and squalene. Additionally, the phenylpropanoid pathway shows the synthesis of phenolic compounds such as Cannflavin A and apigenin from phenylalanine-derived intermediates like p-coumaroyl-CoA and feruloyl-CoA.

*Cannabis sativa* L., a short-day photoperiodic species, is cultivated for an array of diverse purposes encompassing fiber production, food sources, medicinal applications, and recreational consumption [1]. This botanical species is taxonomically bifurcated into *C. sativa* and drug-type varieties, a classification predicated on their utilization purpose and the distinctive chemical profiles of their inflorescences [1]. In the realm of pharmaceutical applications, *C. sativa* is predominantly cultivated for its prolific production of secondary metabolites, which include cannabinoids (CBD, CBG, THC, CBDA, THCA, CBGA, etc.) terpenes, and flavonoids [2]. Notably, these bioactive compounds predominantly accumulate within the glandular trichomes located on the female flowers and the proximate sugar leaves. The biosynthetic pathways responsible for the synthesis of these secondary metabolites can be delineated into three principal metabolic routes: the polyketide synthase (PKS) pathway, the mevalonic acid (MVA)–cytosolic mevalonate (MEV) pathway, and the Palstidial methylerythritol phosphate (MEP) pathway [1,3]. Specifically, the PKS pathway is instrumental in the production of Olivetolic acid (OLA), which serves as the precursor molecule for phytocannabinoid biosynthesis (Figure 1) [1]. Within the glandular trichomes, which are predominantly found on the unfertilized female inflorescences, the synthesis and accumulation of cannabinoids and terpenes occur [1,2]. These inflorescences, therefore, represent the most pharmaceutically valuable segment of the plant, owing to their high concentrations of bioactive secondary metabolites. This intricate biosynthetic process underscores the significance of unfertilized female inflorescences in the pharmacological utility of *C. sativa* [1,2].

The life cycle of industrial *C. sativa* can be delineated into four distinct stages: (i) germination/cloning, (ii) vegetative growth, (iii) flowering and seed formation, and (iv) senescence [4]. The maximization of marketable biomass production, particularly the mature, unfertilized female inflorescences, constitutes a primary objective in the cultivation of cannabis [1,2]. To propel this nascent industry forward, it is imperative to concentrate research efforts on cultivars exhibiting minimal variability in growth metrics, biomass yield, seed quality, and secondary metabolite profiles [5]. Comprehending the intricate interplay between genotype and environmental variables, including photoperiod, in the phenology of *C. sativa* varieties is essential for optimizing biomass production, enhancing product potential, and minimizing nutrient inputs to achieve sustainable agricultural practices [5]. Although the concentrations of secondary metabolites are predominantly dictated by plant genetics, environmental conditions exert a significant modulatory influence [5]. Thus, agricultural productivity can be substantially augmented through the meticulous manipulation and optimization of environmental parameters to favor desired yield outputs [5]. Indoor cultivation, in particular, presents a distinctive advantage, facilitating the rigorous control of environmental factors such as light quality and temperature [6,7]. This controlled environment paradigm stands in stark contrast to traditional outdoor farming, where agricultural productivity is frequently hindered by unpredictable climatic conditions [6,7]. Therefore, the strategic optimization of these environmental variables in indoor cultivation settings holds the potential to substantially elevate yield outcomes [6,7].

Light conditions, encompassing light quality, light intensity, and photoperiod, exert a profound influence on plant growth and development, with light quality representing the most intricate factor [8]. Indeed, plant responses to these varied light conditions are mediated by a diverse array of photoreceptors. The light spectrum can, both directly and indirectly, impact photosynthesis by modulating leaf structure, size, senescence rate, photosynthate transport, stomatal conductance, transpiration, gas exchange, chlorophyll content, plant height, stomatal opening, circadian rhythm, photomorphogenesis, flowering time, and chlorophyll biosynthesis [9]. Extensive research has elucidated the impact of light cycles and environmental conditions on plant development from germination to senescence, with numerous studies examining the correlation between photoperiod and flowering patterns across different plant families by regulating flowering-induced genes [10,11]. Light stands as a pivotal environmental factor for plant growth and development, with stressful conditions capable of inducing the excessive production of reactive oxygen species (ROS) (Figure 1) [12,13]. ROS can wreak havoc on cellular components, including carbohydrates, lipids, proteins, and DNA, potentially leading to plant death. To combat this onslaught, plants deploy a sophisticated arsenal of enzymatic and non-enzymatic antioxidants to regulate ROS production [14].

Photosynthetic carbon fixation, which hinges on light energy, is acutely responsive to light intensity at specific wavelengths. Photoperiodism governs plants’ developmental responses to the daily light-dark cycle, while photomorphogenesis orchestrates the profound effects of light quality on plant development and physiology [5,12,13,14]. Secondary metabolism in plants is heavily influenced by external factors such as light intensity, light spectrum, day length, mineral nutrition, plant architecture, and temperature. In *C. sativa*, secondary metabolites serve critical photoprotective roles. Evidence reveals that light stress or manipulation can dramatically alter the plant’s metabolomic composition. Adjusting wavelength composition can also significantly impact phytohormone activity, stimulating flowering, inhibiting stem elongation, or reducing plant height. Chloroplasts, the epicenters of light sensing, dynamically alter their ultrastructure in response to varying photoperiods. The quality, quantity, and length of light are paramount, influencing the excitation of Photosystem I (PSI) and Photosystem II (PSII), the linchpins of photosynthesis (Figure 1) [5,12,13,14].

Moreover, light parameters can bolster plant resistance to drought and salt stress, and recent evidence underscores the impact of light duration on plant responses to pathogen infection. Light manipulation holds immense potential, capable of regulating various aspects of insect life history, presenting a powerful tool for insect pest management in controlled environments [5,12,13,14]. The yield quantity and quality of inflorescences in *C. sativa* can be markedly enhanced within controlled environment cultivation systems, where all environmental parameters and cultivation practices are meticulously regulated. Indoor cultivation often becomes indispensable due to unsuitable climatic conditions, stringent regulatory restrictions, or a synergistic effect of both factors [5,15]. Among the myriads of factors influencing successful indoor cannabis cultivation, light—encompassing photoperiod, quality, and intensity—remains paramount [5,15]. To ensure uniform environmental conditions and produce a consistent product, there is a burgeoning trend towards cultivating cannabis in controlled indoor environments. Within these ‘in-door’ facilities, artificial lighting systems provide the requisite light, with fixtures tailored to vary in intensity and spectral composition [5,15].

*Cannabis*, cultivated for both recreational and medicinal purposes, is recognized as a high-value crop predominantly grown within controlled environment production facilities, such as greenhouses utilizing natural light or growth chambers and vertical farms devoid of natural light [5,15]. These cultivation setups facilitate consistent, year-round production. The propagation, vegetative growth, and flowering stages of indoor cannabis production exhibit distinct photoperiod and light-intensity requirements [5,15]. Optimal post-vegetative stage morphology is contingent upon the production system employed by cultivators, which encompasses variables such as the length of the vegetative stage, planting density, substrate and root zone volume, and the type of trellising system implemented during flowering [5,15]. Nevertheless, the overarching objective is to ensure high transplant success rates and robust vegetative growth. Maintaining rigorously controlled conditions is indispensable for the consistent production of medical cannabis, particularly concerning inflorescence yield and the concentrations of secondary metabolites [5,15]. These controlled environment cultivation systems not only facilitate the optimization of growth conditions but also ensure the production of a high-quality, uniform product, thus meeting the stringent demands of both medicinal and recreational cannabis markets [5,15].

In this review, we conducted a comprehensive examination of recent research focused on the impact of indoor light on the growth, development, and secondary metabolite production in *C. sativa*, situating our analysis within the broader context of light signaling in plant physiology. Our findings underscore pivotal areas where the intricate interaction between light, and *C. sativa* necessitates further elucidation to achieve a comprehensive understanding of its underlying mechanisms.

## 2. Role of Light in Plant Growth, Development, and Secondary Metabolism: A Background Story of Mechanism

Light, a critical environmental factor, influences plant growth, development, and metabolism, functioning in both photosynthetic and signaling capacities for plant morphogenesis [16]. Variations in light quality (spectrum), intensity, and duration catalyze critical physiological and biochemical reactions, dramatically shaping plant form and function (Figure 1) [16]. Plants have evolved a sophisticated circadian clock to synchronize various processes with the light/dark cycle, and metabolic reprogramming in response to light is crucial for optimal plant growth and development [16]. Understanding these processes is pivotal for improving crop production efficiency and agricultural productivity [16].

Light intensity, spectrum (280–800 nm), and day length significantly affect reactive oxygen species (ROS) formation during photosynthetic electron transport (Figure 1), influencing the cellular redox state crucial for metabolic adjustments that enable plant survival under various environmental conditions [17,18]. The dynamic shifts in light conditions, both spatial (latitude, altitude) and temporal (daily, seasonal), are compounded by fluctuations in temperature, water supply, and stress factors. Consequently, light-governed redox control profoundly influences core metabolic pathways (carbon, nitrogen, amino acids, sulfur, lipids, and nucleic acids) and secondary metabolism (terpenoids, flavonoids, alkaloids) (Figure 1) [17,18].

ROS accumulation is particularly intense near electron transport chains, necessitating precise, compartment-specific redox adjustments [17,18,19]. Within chloroplasts, excess ROS emerges near thylakoid membranes during photosynthesis, specifically in the electron transport between photosystems II and I (Figure 1). In mitochondria, ROS accumulate around inner membranes during respiration [17,18,19]. The antioxidant system is crucial in maintaining optimal ROS levels, safeguarding cells from oxidative devastation under various environmental stresses [15,17,18]. Dominant antioxidants include the ascorbate–glutathione cycle, α-tocopherol, carotenoids, flavonoids, thioredoxins (TRXs), peroxiredoxins (PRXs), superoxide dismutase (SOD), catalase (CAT), peroxidase (POD), and glutathione *S*-transferases (GSTs) [15,17,18]. The synergistic roles of ROS and antioxidants in light-dependent redox regulation are critical for plant adaptation to environmental changes. The strategic metabolic engineering of light signaling intermediates to modulate secondary metabolite accumulation is essential for maximizing plant growth and development [15,17,18] (Figure 1). The light-induced synthesis of photoprotective secondary metabolites is indispensable [17,18,19].

The spectral regulation of metabolism is of utmost importance due to the precise absorption maxima of chlorophylls and the acute sensitivity of photoreceptors (Figure 1). Plants employ sophisticated photoreceptors to precisely adapt, to environmental light cues, drive extensive transcriptome reprogramming, and induce developmental and physiological changes (Figure 1) [16,20]. Light perception, which is crucial for photomorphogenesis and metabolism, involves specific photoreceptors (Figure 1) distinct from photosynthetic pigments that operate independently of photosynthesis [16,20]. Five formidable classes of photoreceptor proteins have been identified in plants: phytochromes, cryptochromes, phototropins, the ZEITLUPE (ZTL)/FLAVIN-BINDING KELCH REPEAT F-BOX 1 (FKF1)/light oxygen voltage (LOV) KELCH PROTEIN 2 (LKP2) complex, and UV RESISTANCE LOCUS8 (UVR8) [16,20]. Phytochromes, which are essential for red and far-red light responses, exist in two states: Pr, absorbing red light (650–670 nm), and Pfr, absorbing far-red light (705–740 nm) [16,20]. Pr converts to the active Pfr upon absorbing red light, and Pfr reverts to Pr upon absorbing far-red light. Phototropins, membrane-bound and activated by blue light, are critical in photosynthesis, regulating chloroplast movement, stomatal dynamics, and optimizing carbon dioxide and water exchange [16,20,21]. The ZTL/FKF1/LKP2 complex, sensitive to blue and UV-A wavelengths, governs circadian rhythms and flowering [16,20,21]. UVR8 receptors, detecting UV-B radiation, mitigate its harmful effects by inducing gene expression linked to antioxidant production. They also regulate vital plant responses, such as stomatal behavior and chlorophyll balance [16,20,21]. These advanced systems allow plants to adjust their growth and development, ensuring survival and optimal performance in dynamic light environments [16,20,21,22,23,24].

Photoreceptor-mediated light perception initiates a signaling cascade that markedly changes gene expression and influences key plant physiological responses [16,20,21]. In Arabidopsis, a staggering 20% of genes are regulated by light, with the bZIP transcription factor ELONGATED HYPOCOTYL 5 (HY5) emerging as a pivotal regulator in light-mediated development and metabolism. HY5 integrates light signals with hormonal and nutrient pathways, while Phytochrome Interacting Factors (PIFs) serve as strong negative regulators of this pathway. The E3 ubiquitin ligase CONSTITUTIVELY PHOTOMORPHOGENIC1 (COP1) controls these transcription factors by targeting them for degradation. HY5’s versatility spans light, hormone, and stress signaling, operating downstream of phytochromes, cryptochromes, and UVR8 to regulate gene expression [16,20,21]. Its interactions with SUPPRESSOR OF PHYA (SPA) proteins and other transcription factor families highlight its central role in light signaling. The transcription of light-regulated genes significantly impacts plant growth, development, and seasonal adaptation [16,20,21]. Hormones such as auxin, ethylene, jasmonic acid, gibberellic acid, and abscisic acid interact with light signaling pathways, influencing both vegetative and reproductive stages [16,20,21,25,26]. Recent advancements have clarified hormone signaling (Figure 1) mechanisms, revealing how light perception profoundly affects hormonal regulation in processes like germination, flowering, and morphogenesis [16,20,21].

The circadian clock aligns plant physiology and development with daily and seasonal environmental changes, controlling processes such as photosynthesis and stomatal movements [17,26]. Chromatin regulation creates rhythmic gene expression networks that are active at specific times and managed by the clock through feedback loops, involving MYB transcription factors (Figure 1) [17,26]. Shade avoidance, which is crucial for survival, is linked to hormone signaling, such as the auxin-mediated elongation of hypocotyls and petioles [17,25,26,27]. Plants detect shading from neighboring vegetation through dramatic shifts in the red-to-far-red (R–FR) light ratio, sensed by phytochromes.

The response cascade involves hormones like gibberellins (GA), ethylene (ET), auxin, Brassinosteroids, cytokinins (CK), and jasmonic acid (JA) (Figure 1) [17,25,26,27]. Auxin plays a pivotal role in elongation phenotypes and its pathway is tightly regulated when sensing nearby plants. Auxin-related genes dominate a significant portion of the shade avoidance transcriptome [17,25,26,27]. The regulation of apical dominance is another critical aspect governed by endogenous auxins and cytokinins. These growth regulators are instrumental in nutrient diversion, gene expression modulation of axillary bud growth, and fine-tuning the auxin/cytokinin ratio (Figure 1) [17,25,26,27]. The synthesis of auxins at the plant apex and their strategic transport to axillary buds play a crucial role in maintaining apical dominance [17,25,26,27]. These mechanisms are key to understanding plant growth and development in response to light signals and underscore plant adaptability and resilience [17,25,26,27].

Synchronized flowering is crucial for plants, particularly in regions with significant seasonal variation and those requiring cross-pollination [26,28]. Because flower and seed development are energy-intensive processes, they must occur under optimal conditions. Therefore, flowering is meticulously regulated by environmental factors such as day length and temperature, as well as internal signals, including hormonal levels, sugars, and plant age (Figure 1) [26]. Phytochromes A and B (Phy A and Phy B) and cryptochrome 2 (cry2) are crucial photoreceptors that mediate photoperiodic flowering [26]. Different hormones further influence this process. Understanding how these environmental cues and hormonal signals interact is key to deciphering the mechanisms that govern the timing of flowering in plants [26].

Light conveys critical environmental information to plants influencing defense hormone signaling, resource allocation, and adaptation [27]. Jasmonate (JA) and salicylic acid (SA) are powerful defensive phytohormones, and their pathways are profoundly influenced by light, impacting plant defense, growth, and development (Figure 1). Photoreceptors and transcription factors in phototransduction are integral to these hormones’ signals [27], significantly advancing the use of artificial lighting for crop growth and disease management in greenhouses. Jasmonate orchestrates the accumulation of vital defense-related metabolites and proteins, along with MYB, and trichome-specific transcription factors that play crucial roles in UV light signaling and terpenoid production (Figure 1). The light spectrum significantly controls terpenoid biosynthesis and determines plant quality, including aroma, flavor, color, and medicinal properties [27]. Light conditions such as blue, red, and far-red light enable HY5 to drive secondary metabolite production. In Artemisia annua, HY5 binds to specific transcription factor genes, dramatically upregulating their expression and fueling polyphenol, terpenoid, and alkaloid biosynthesis [27,28,29,30,31].

Artificial lighting, such as high-pressure sodium (HPS) lamps, light-emitting diodes (LEDs), and fluorescent lamps, are crucial for indoor cannabis cultivation to meet the light energy needs of the plants. Over the past decade, the use of LEDs in horticulture has skyrocketed due to their overwhelming advantages over traditional light sources (Figure 1). LEDs boast unparalleled energy efficiency, minimal heat emission, and exceptional longevity. They offer superior efficacy and significantly lower operating costs compared to HPS lamps [5,16]. They can emit specific wavelengths, enabling groundbreaking research on the effects of different wavelengths on secondary metabolite production, although data on the effects of varying radiation intensities and qualities on cannabis secondary metabolite composition are limited and sometimes contradictory [5,16]. LED adoption has revolutionized horticultural practices by enabling growers to customize the light spectrum and intensity for different crops and developmental stages, thereby enhancing production schedules, crop yield, and quality. Traditionally, high-intensity discharge (HID) lamps like metal halide (MH) and high-pressure sodium (HPS) lamps have dominated greenhouses and growth chambers (Figure 1) [5,16]. However, the exploration of monochromatic LED systems as replacements for traditional light sources is underway, particularly in space greenhouses, to optimize crop production and quality through meticulously designed light recipes. By combining LEDs of various colors, growers can create a tailored light spectrum at the desired intensity, effectively modulating different plant functions [5,16]. This provides a powerful tool for controlling plant growth and photomorphogenesis [5,16]. These advancements in LED technology hold immense potential for enhancing horticultural practices both on Earth and in space. They enable precise control over the light environment to meet the specific needs of plants at various stages of development [5,16]. Matching LED wavelengths to plant photoreceptors can maximize output and modify plant shape and metabolism, making LEDs indispensable for sustainable production and photomorphogenesis research [5,16].

## 3. Light Effect on Growth and Development of *C. sativa*

### 3.1. Light Spectrum in Cannabis Growth, Development, and Metabolism

The pivotal role of light’s quality (spectral) composition in plant growth and physiological functions has been paramount since the dawn of photosynthesis research [32]. Recently, this field has exploded with intensity due to revolutionary advancements in computer-controlled lighting systems and the sophisticated documentation of photosynthetic activity. These strides are fortified by groundbreaking insights into the molecular mechanisms of photo regulation, governed by an array of photoreceptors and pigment systems [32].

The profound impact of varying light spectra is rooted in (i) the precise activation of distinct photoreceptors, (ii) the disparate efficiency of spectral components in driving robust photosynthesis, and (iii) the varying depth of spectral penetration into plant foliage (Figure 1) [32]. These dynamic factors orchestrate chloroplast movements, optimize light capture, and elevate photosynthetic capacity and metabolic processes [32]. Blue light, for example, dramatically boosts the biosynthesis of chlorophyll *a*, and alters the Chl *a*/*b* ratio by regulating key gene expressions [33]. The synthesis and catabolism of proline, a critical compound for stress adaptation and redox balance, are also profoundly influenced by light quality [17,33,34]. Carbohydrate concentration, a vital indicator of cell division activity, is powerfully correlated with the intensity of photosynthetically active radiation (PAR) (Figure 1) [35,36]. Blue light dramatically enhances total soluble carbohydrates (TSC) and starch accumulation, while red–blue light treatments significantly boost both fresh and dry biomass in plants. Blue light (400–500 nm) typically results in smaller, more compact leaves and shorter stems. Greenlight (510–585 nm) effectively mitigates lower leaf loss, whereas red light (620–700 nm) can inhibit flowering [37]. In stark contrast, a combination of red and far-red light (700–780 nm) forcefully triggers flowering and elongates leaves and stems [37,38].

A low red-to-far-red light ratio deactivates phytochrome b, spikes gibberellin (GA) levels, and obliterates DELLA proteins. Despite its detrimental impact on quantum yield, supplemental UV-A radiation can dramatically elevate photosynthetic rates and biomass accumulation in *C. sativa* [37,39]. Blue light profoundly influences chlorophyll biosynthesis, plant height, and stomatal opening while driving the accumulation of phenylpropanoid-based compounds without altering the plant morphology of the *C. sativa* plant [40]. Red light, however, drastically transforms plant morphology and physiology, vigorously stimulating stem growth and flowering but failing to enhance secondary metabolism [40]. The precise balance of blue to red light is crucial for maximizing growth, pigment production, and antioxidant capacity in vegetable plants, although this balance is species-specific [40]. Plants exhibit species-specific responses to light stress, mediated by a diverse array of photoreceptors. Red light markedly boosts stomatal conductance, net photosynthesis, intercellular CO_2_ levels, and transpiration rates, while blue light suppresses these parameters by inducing stomatal closure [40]. A combination of red and blue light can severely stifle growth and photosynthesis in *C. sativa*, increasing MDA and proline content, and protecting cellular organelle membranes (Figure 1) [40].

The spectrum of light wields profound power over photosynthesis, directly and indirectly altering leaf structure, size, senescence rate, photosynthate transport, stomatal conductance, and chlorophyll composition [41]. Blue light, in particular, exerts a significant influence on plant height, stomatal opening, circadian rhythms, photomorphogenesis, flowering time, and chlorophyll biosynthesis [42]. Despite the widespread practice of indoor *C. sativa* farming, the precise effects of red and blue LED lighting on CBD (cannabidiol) synthesis remain shrouded in uncertainty [43]. Uncovering the optimal red and blue LED ratios could revolutionize CBD yield in indoor *C. sativa* cultivation [8]. The impact of LED light wavelengths on *C. sativa* growth and cannabinoid synthesis is nothing short of transformative. Experiments involving six distinct LED treatments (with varying red-to-blue light ratios and photosynthetic photon flux density) revealed that LED2 (R/B 1.61/1) and LED5 (R/B 16.8/1) effectively sustained plant height, stem diameter, and leaf numbers compared to high-pressure sodium lighting. LED2 dramatically boosted CBD content in both leaves and flowers, while LED5 significantly enhanced CBD levels in leaves. These findings underscore that specific red and blue LED ratios can drastically influence *C. sativa* growth and cannabinoid synthesis, positioning LED2 and LED5 as prime candidates for maximizing CBD yield [8]. In the realm of pest management, the objective is to obliterate yield reduction and uphold optimal CBD/THC (tetrahydrocannabinol) ratios [44].

Manipulating light spectra in indoor cannabis cultivation has revealed its formidable potential to impact plant growth and pest interactions. Increased blue light has been shown to amplify bud yield compared to white light, whereas red–blue light treatment results in towering plants with diminished leaf–stem dry mass and bud yield. Herbivory, meanwhile, slashes bud yield and CBD/THC concentration (Figure 1) [44,45]. Light quality also affects pest biology, indicating its untapped potential as a pest management weapon. For example, aphid-induced damage triggers salicylic acid-mediated defenses, escalating the production of secondary metabolites such as terpenes and cannabinoids (Figure 1) [44,45], which possess lethal insecticidal properties [44]. In the absence of aphids, ramped-up blue light supercharges bud mass and CBD content in *C. sativa*. Conversely, when aphids are present, white light initially curtails their growth and survival, but red–blue light may bolster cola production and sustain yield quality. However, these outcomes are intricately context-dependent and laden with trade-offs. Employing light quality and herbivory as management tactics harbors colossal potential but demands rigorous exploration for integrated pest management in controlled environments (Table 1) [44]. These groundbreaking discoveries offer potent new tools for optimizing *C. sativa* cultivation and executing devastatingly effective pest control.

The symbiotic relationship between mycorrhiza and plants is a dominant force in the plant kingdom, thriving across ecosystems and captivating scientific inquiry for over two centuries. Light is not merely a growth factor; it is a catalyst for plant metabolism, igniting a cascade of biochemical transformations that shape every facet of plant development—from morphology to pigment synthesis, phenolic compound production, and hormone regulation [66]. Blue light stands out as a powerhouse, crucial for the large-scale, sustainable production of industrial *C. sativa* [66,67]. Chloroplast pigments, the vibrant bioactive compounds inherent in nature, are essential for their coloration and potent antioxidant effects. Carotenoids, in particular, are indispensable for shielding cells from oxidative onslaughts (Figure 1) [61,67]. Phenolic compounds, with their formidable radical-scavenging capabilities and protein interactions, are key players in antioxidant defense [68]. In groundbreaking research, various biopreparations, including vesicular-arbuscular mycorrhiza (VAM), *Azotobacter chroococum*, and *Trichoderma* spp., were inoculated on *C. sativa* seeds and/or substrates, then exposed to blue and white light. The findings were stark: blue light dramatically undermined bio-preparation treatments (Figure 1), leading to significantly lower morphological parameter values compared to controls (Table 1) [61]. Yet, blue light-induced higher leaf pigment levels. Crucially, the type of light did not alter the antioxidant properties, including DPPH(2,2-diphenyl-1-(2,4,6-trinitrophenol) hydroxyl), FRAP (ferric reducing antioxidant power), flavonoids, total flavanol content, or phenolic acids (Figure 1) [61]. Biopreparation treatments also did not markedly influence leaf pigment content (Chl a, Chl b, and carotenoids) or phenolic and flavanol content (Figure 1). These revelations demand further investigation, particularly over prolonged periods or under field conditions, to unlock the full potential of bio-preparation treatments with beneficial microorganisms on the Finola *C. sativa* cultivar [61]. The implications are profound, offering a path to revolutionize *C. sativa* cultivation with enhanced growth, resilience, and biochemical richness [61].

Monochromatic blue light stands as a formidable tool for enhancing secondary metabolite production, though it can significantly impair growth traits like inflorescence size. Strategic, intermittent supplementation with blue light at the terminal stage of the growth cycle [30] shows great potential for dramatically elevating THC levels without substantially diminishing biomass [58]. Dichromatic LED lighting emerges as a groundbreaking alternative, mitigating the adverse effects of pure blue light while sustaining secondary metabolite levels comparable to conventional HPS lighting. An innovative study scrutinized the impact of six distinct light spectra from dichromatic B LED lights on the growth traits and secondary metabolite profiles of *C. sativa* (Babbas Erkle Cookies’) [58]. These spectra included blue (430 nm), red (630 nm), rose (430 + 630 nm, 1:10 ratio), purple (430 + 630 nm, 2:1 ratio), and amber (595 nm) LEDs and an HPS control. Blue light treatment yielded the highest THC concentration but the lowest THC per plant, whereas the HPS treatment produced the highest THC per plant. Blue light also markedly increased cannabigerol (CBG) and terpene concentrations but had a limited effect on cannabidiol (CBD) biosynthesis (Table 1) [58]. These groundbreaking findings empower producers to tailor spectral designs to specific production objectives, thereby optimizing cannabis production efficiency and slashing operational costs. Future research should be on light qualities richer in the amber spectrum and fine-tuning blue light proportions. Hybrid grow rooms integrating HPS and LEDs could substantially boost light intensity and profitability. *C. sativa* seedlings were meticulously evaluated under 11 light treatments within an advanced aeroponics system, assessing morphological traits, photosynthetic pigments, and osmolytes (Figure 1) [58].

Treatments L10 (R4/B2) and L11 (R2/B2/G2) excelled in leaf and node numbers. Treatments L2 (white), L3 (R8), and L5 (R7) achieved superior leaf length and width. Exceptional shoot lengths were noted in L3, L6 (R6), and L9 (R6), while root development flourished in L1 (natural light), L5, and L9. L3 exhibited the highest levels of chlorophyll a, chlorophyll b, and photosynthetic quantum yield (Fv/Fm) (Table 1) [37,69]. Hierarchical clustering analysis revealed that higher leaf and node numbers resulted in bushier plants with shorter shoots and longer roots. There was a stark negative correlation between photosynthetic traits and osmolytes with root length (Figure 1). Treatments L4, L6, L8 (R5/B2), and L11 displayed increased node numbers and osmolyte content—proline, ascorbic acid, total soluble carbohydrates (TSC), and sucrose—indicating greater branching, inflorescences, and cannabinoid accumulation [37,69] (Table 1). This pioneering study provides a crucial foundation for future research, steering the optimization of light compositions to cultivate *C. sativa* with ideal phenotypes. Notably, incorporating green light into other spectra enhanced osmoprotectant molecules compared to red and blue combinations alone, heralding a new era in precision agriculture for *C. sativa* cultivation.

The research underscores the significant impact of light quality on the morphophysiological traits of industrial *C. sativa* [70]. A key study investigated the effects of different light spectra—white (WL), blue (BL), red (RL), and a 50:50 red–blue mix (RBL)—on *C. sativa*’s morphology, gas exchange, and antioxidant capacity. The results were remarkable: BL increased shoot fresh biomass by 15.1%, shoot dry biomass by 27.0%, leaf number by 13.7%, stem diameter by 10.2%, root length by 6.8%, and chlorophyll content by 7.4% compared to WL. Additionally, BL significantly improved net photosynthesis, stomatal conductance, and transpiration while lowering lipid peroxidation and antioxidant enzyme activities. Conversely, RL and RBL treatments notably decreased plant biomass and gas exchange parameters while elevating antioxidant enzyme activity [40] (Figure 1). These groundbreaking findings solidify blue light’s role in sustainable, large-scale *C. sativa* production, though further research is crucial to unravel the precise mechanisms through which light governs *C. sativa* growth and development.

The rooting of stem cuttings is a cornerstone of plant propagation, profoundly influenced by endogenous auxins, particularly indole-3-acetic acid (IAA) [71,72]. IAA, produced in the shoot apical meristem or young leaves, is transported to the base, sparking cell division and adventitious root formation (Figure 1) [71,72]. Light is critical in this process, supplying energy for photosynthesis and signaling photomorphogenesis. However, high radiation can devastate auxin levels [73]. Far-red light (700–800 nm) stands out by boosting endogenous auxin accumulation via phytochromes, igniting shade avoidance responses, and enhancing auxin synthesis and stem elongation [73]. A revolutionary study on medicinal cannabis delved into the effects of different light spectra (blue, red, far-red) on the adventitious rooting of stem cuttings and their relationship with endogenous auxin and carbohydrates [56]. The study’s revelations were profound: far-red light dramatically improved rooting in one of two experiments, particularly when combined with red or red-blue light [46] (Table 1). Notably, far-red light applied during the initial rooting stage was sufficient to enhance rooting without triggering excessive stem elongation. While the positive effects of far-red on auxin and carbohydrate concentrations likely underline the improved rooting, the study did not find a direct correlation between these factors and rooting success (Figure 1) [46]. These powerful findings underscore the potential of optimizing light spectra for both industrial *C. sativa* production and medicinal cannabis propagation, heralding a new era of unparalleled efficiency and effectiveness in cultivation practices.

Until recently, clonal cannabis propagation primarily relied on fluorescent lighting [74]. The shift to LEDs marks a revolutionary advancement, allowing cultivators to implement precise spectral treatments that enhance rooting and drastically cut energy consumption [74]. A groundbreaking study explored the effects of various LED combinations—blue (B), red (R), ultraviolet-A (UVA), and phosphor-converted white (W)—against a fluorescent (F) control on vegetative stem cuttings of ‘Gelato-27’, ‘Grace’, and ‘Meridian’. Rooting for 15 days under each lighting treatment, maintaining a PPFD of 200 µmol m^−2^ s^−1^ with a 16 h photoperiod, yielded remarkable results: while spectrum treatments did not affect rooting percentages, root index values soared under B+UVA compared to F (Table 1). Moreover, root dry weights in B, B+UVA, B50, and F treatments surpassed those in the W treatment [55]. At the plug stage’s end, no significant effects on chlorophyll content index were noted. However, cuttings under B exhibited thicker stems than those under BR and W, and F showed the lowest percentage of new aboveground growth.

Blue (400–500 nm) and red (600–700 nm) wavelengths in horticultural LEDs deliver the highest photosynthetic quantum yield without triggering abnormal responses [55] (Figure 1). In *C. sativa*, secondary metabolites accumulate in the trichomes of female flowers and sugar leaves, synthesized through metabolic pathways: the PKS pathway produces Olivetolic acid (OLA) and the MVA/MEV and MEP pathways produce dimethylallyl diphosphate (DMAPP) and geranyl diphosphate (GPP). GPP, combined with OLA, forms cannabigerol acid (CBGA), the precursor to all cannabinoids, linking monoterpene and cannabinoid biosynthesis (Figure 1). These findings highlight the transformative potential of optimizing light spectra and metabolic pathways to enhance cannabis cultivation, heralding a new era of efficiency and potency [75,76].

Light, temperature, and various stresses play pivotal roles in directing the allocation of isoprenoid precursors between the MEP and MEV/MVA pathways by modulating enzyme expression. Enhanced light intensity significantly represses the MEV/MVA pathway while stimulating the MEP pathway [77]. Terpene synthases (TPSs) are critical in converting these precursors into specific mono- and sesquiterpenes, with their activity being profoundly influenced by light. Notable TPSs include TPS-a (caryophyllene and humulene), TPS g (linalool and nerolidol), and TPS-b (β-myrcene, limonene, α-pinene) (Table 1) [75,76]. Despite significant advances in understanding TPS genetics, predicting terpene profiles remains challenging due to external factors such as light, temperature, nutrition, and stress. The developmental stage of the plant also significantly impacts terpene and cannabinoid distribution, peaking in reproductive parts during flower maturity [57,75,76,77]. High R/FR light spectra activate phytochrome (Pfr), enhancing MEP pathway enzyme expression and increasing monoterpene and CBDA concentrations in cannabis buds. Different light spectra markedly influence CBDA and terpene concentrations, with variations reflecting interactions between plant morphology and chosen light spectra (Figure 1).

Techniques like strategic pruning and defoliation can greatly enhance light-use efficiency and optimize flower exposure [57]. A groundbreaking study on a CBD-dominant *C. sativa* genotype, FINOLA, examined the effects of long (far-red) and short-wavelength (blue, UV-A, UV-B) radiation on morphology, inflorescence yield, and secondary metabolites. This study compared LOW R/FR (ratio of 1) and HIGH R/FR (ratio of 11) treatments and various blue, UV-A, and UV-B radiation levels. The LOW R/FR treatment increased plant height but reduced inflorescence yield, while the HIGH R/FR treatment significantly boosted CBD, THCVA (tetrahydrocannabivarin acid), CBGA (Cannabigerolic acid), and terpene concentrations (Figure 1) [50]. Short-wavelength radiation treatments did not affect yield or morphology but increased THCVA concentrations, with UV-B specifically raising myrcene levels. These findings underscore the transformative potential of manipulating the R/FR ratio and incorporating short-wavelength radiation to fine-tune cannabis morphology, yield, and secondary metabolite profiles. Mastery of light spectrum manipulation stands as a powerful tool for optimizing cannabis cultivation, paving the way for unprecedented advancements in yield quality and production efficiency [50].

The adaptability of *C. sativa* through significant physiological and biochemical changes was rigorously studied under 10 different LED light spectra (a combination of red, blue, green, far-red, UV, and natural light) using an advanced aeroponic system [32]. The R7/B2 light treatment propelled cannabidiol (CBD) levels to unprecedented heights, coupled with an increased photosynthetic rate and substantially lower levels of reactive oxygen species, total phenols, flavonoids, DPPH radical scavenging capacity, and antioxidant enzyme activities (Figure 1) [32]. Under these conditions, THC and CBD showed minimal stress responses, while THCA emerged as the primary stress marker, closely followed by CBDA. Notably, CBD concentrations surged under treatments L4 (R7), L5 (R7), and L8 (R5/B2/G1), with THC levels consistently surpassing those under natural light across all spectra. CBDA (cannabidiolic acid) synthesis peaked in L3 (R8), L4 (R7/B2), L6 (R6/B2/G1), and L8. The L4 treatment delivered staggering concentrations of CBD, CBDA, THC, and THCA while minimizing stress markers. In stark contrast, L6 resulted in lower CBD and THC but higher THCA and CBDA, alongside intensified stress response activity [62]. Green light emerged as a pivotal factor in CBD and CBDA synthesis, with far-red and UV-A light displaying complex, nuanced effects. Photosynthetically active radiation spans 400 to 700 nm, with red (600–700 nm) and blue (420–450 nm) wavelengths wielding the most significant influence due to their optimal absorption by chlorophylls a and b, complemented by carotenoid absorption (Figure 1) (Table 1) [62].

Recent revelations suggest that a full spectrum (white light) dramatically outperforms monochromatic red and blue lights in promoting plant growth and functionality [62]. LED light spectra profoundly impacted every measured morphological, physiological, and chemical parameter [62] (Table 1).

The zenith of inflorescence yields was achieved under red and blue light at a 1:1 ratio, with similar success observed at a 1:4 blue/red ratio in two of the three varieties tested [15]. Conversely, white light with a 1:1 blue ratio yielded the lowest output. Light spectrum intricately shaped the chemical profile, with CBGA showing the most robust response, particularly under blue-rich light compared to far-red-rich HPS light [15]. Major cannabinoids like CBDA, THCA, and CBCA (cannabichromene acid) were also significantly affected, although responses were cultivar-specific and less pronounced than for CBGA. Blue light unequivocally promoted compact plant morphology, surpassing the influence of red/blue ratios (Table 1) [15]. The study uncovered cultivar-dependent responses, highlighting genetic variance and unveiling new avenues for breeding cannabis varieties optimized for light-induced responses [15]. Water relations and oxidative stress parameters remained largely unaffected by light quality across all varieties, underscoring the minimal impact of light spectra on *Cannabis* water relations [15]. The full spectrum light significantly enhanced inflorescence yield compared to blue/red light, reinforcing the hypothesis that the light spectrum is a powerful tool for manipulating plant development and cannabinoid profiles, offering unparalleled opportunities to fine-tune cannabis and cannabinoid production (Table 1) [15]. These findings illuminate the transformative potential of strategically optimized light spectra in revolutionizing *Cannabis* cultivation, maximizing both yield and cannabinoid content while effectively managing plant stress responses. This paradigm shift in horticultural practices paves the way for unprecedented advancements in the efficiency and potency of *Cannabis* production.

### 3.2. Effect of Photoperiod Growth and Metabolism of C. sativa

The Earth’s rotation and revolution create profound 24 h cycles of day and night and dramatic annual shifts in temperature and humidity. These cyclical changes, though modest in tropical and subtropical regions where precipitation defines seasons, are monumental in temperate regions where daylight and temperature variations dictate the four seasons. Daylight is an unfaltering beacon, far more predictable than temperature, enabling organisms to anticipate seasonal changes and masterfully orchestrate their biological functions. Photoperiod, the length of the light period over 24 h, is the maestro of plant growth. Trees, for example, halt their growth in autumn as days shorten, foreseeing the harsh winter [10]. Photoperiods and circadian clocks are the bedrock of plant development and adaptation, synchronizing key developmental transitions with optimal conditions, and ensuring ecological fitness and survival [10] (Figure 1).

*Cannabis* plants, often exhibiting short-day photoperiod responses, initiate flowering as daylight wanes. Despite extensive cultivation and breeding creating significant genotypic variations in photoperiodic responses, some cultivars, particularly those bred with equatorial genotypes, are day-neutral or “auto-flowering”, transitioning to flowering based on physiological age rather than photoperiod [60]. A pioneering study examined the photoperiod responses of multiple indoor-grown cannabis cultivars, observing flowering initiation under varying light durations (12 to 15 h). Flowering, marked by the appearance of three pairs of stigmas at the apex of the primary shoot, occurred in all cultivars under photoperiods up to 14 h. This finding demonstrates that certain drug-type cannabis cultivars can achieve strong flowering responses under extended photoperiods, potentially leading to higher yields [60] (Table 1). Future research should investigate the yield and quality effects (e.g., cannabinoid composition) of photoperiods longer than 12 h on indoor-grown cannabis cultivars brought to commercial floral maturity [65]. These astonishing findings underscore the pivotal role of photoperiod in cannabis cultivation, offering a blueprint for optimizing growth conditions to achieve unparalleled yields and superior quality. The strategic manipulation of light duration heralds a new era of cultivation mastery, where cannabis plants can reach their fullest potential.

A groundbreaking study assessed the impact of nine distinct flowering photoperiod treatments on the biomass yield and cannabinoid concentration of three medicinal cannabis varieties [59] (Table 1). The varieties included “Cannatonic”, renowned for its high CBD content, and “Northern Lights” and “Hindu Kush”, both celebrated for their high THC levels. Following an initial 18-day cycle of 18 h light/6 h dark, the nine treatments included standard 12L/12D, a shortened 10L/14D, and an extended 14L/10D photoperiod (Table 1) [59]. The results were astonishing: flower biomass yields soared under the 14L/10D photoperiod across all varieties. However, for the THC-dominant varieties, a static 14L/10D photoperiod led to a significant decline in THC concentration [59]. In stark contrast, the “Cannatonic” variety thrived under the 14L/10D treatments, with CBD concentrations skyrocketing, leading to a monumental 50–100% increase in total CBD yield.

This pivotal discovery dismantles the long-held belief that a 12L/12D photoperiod is optimal for all cannabis lines, revealing that lengthening the light period during flowering can dramatically enhance yields for specific varieties [59]. Cannabinoid composition and abundance are predominantly governed by genetics [78]. The circadian clock gene expression, which follows diurnal oscillation, does not change with day length, causing peak expression times to vary with the seasons [59,78] (Figure 1). As cannabis has been cultivated across diverse latitudes, variations in photoperiod response have been naturally selected, influencing flowering timing and overall performance [59,78] (Table 1). In summary, this trailblazing study elucidated that cannabis varieties exhibit unique responses to photoperiod length changes, rendering the standard 12L/12D photoperiod suboptimal for many strains. Remarkably, cannabinoid yields (grams per plant) can more than double by increasing the photoperiod from 12 to 14 h during flowering, as evidenced by the Cannatonic line, due to significant gains in both flower biomass and cannabinoid concentration [59,78] (Table 1). The 14L/10D photoperiod not only achieved exceptional yield benefits but also did so without incurring additional electricity costs compared to the 12L/12D standard. This makes the 14L/10D photoperiod the superior, all-encompassing treatment, ideal for mixed cultivation and untested varieties [59,78] (Table 1).

To enhance growth, *C. sativa* is frequently subjected to extended photoperiods or continuous light, which can induce photooxidative damage and necessitate substantial adjustments in photosynthetic processes [56]. Chloroplasts, the primary light-sensing organelles, dynamically alter their ultrastructure in response to varying photoperiods. Plants are categorized by their photoperiodic responses: long-day, short-day, and day-neutral. Beyond flowering, photoperiod profoundly affects daily photosynthesis, growth, and starch metabolism. The quality and intensity of light are paramount for the activation of PSI and PSII, essential components of photosynthesis [56] (Table 1). Continuous light exposure can impair photosynthesis due to starch accumulation in *C. sativa* leaves, leading to a deficit in electron acceptors and the production of reactive oxygen (Table 1) species (ROS), resulting in significant oxidative damage, known as photooxidative stress [56]. Compounds such as carotenoids and phenyl lipids in the thylakoid membranes serve as the first line of defense against ROS (Figure 1) [56].

A pioneering study employed chlorophyll fluorescence, lipid peroxidation (TBARS), and FT-IR spectroscopy to examine two cannabis cultivars under white (W) and purple (P) light with varying photoperiods (16/8, 20/4, and 24/0). The 16/8 photoperiod, irrespective of light type, was the most beneficial, yielding the highest physiological efficiency and lowest TBARS content, indicative of minimal thylakoid membrane damage (Table 1) [68]. *C. sativa* adapts using highly efficient strategies based on the light type and photoperiod duration. White light facilitated greater dissipation of excess light, reducing the burden on PSI [68]. The P20/4 treatment exhibited effective energy dissipation and cyclic electron transport around PSI, indicative of robust repair mechanisms. Even under continuous light (24/0), P (photoperiod) 24/0 maintained efficient electron transport, positively influencing photosynthetic reactions despite noticeable thylakoid membrane damage [68] (Table 1). These findings underscore the critical importance of optimizing light conditions and photoperiods to enhance photosynthetic efficiency and mitigate oxidative damage in *C. sativa* cultivation. This paradigm shift in light management has the potential to propel *C. sativa* growth and productivity to unprecedented levels, heralding a new era of agricultural excellence [56].

A meta-analytic approach to determine when growers should switch photoperiods to optimize *C. sativa* floral biomass and cannabinoid content. Floral biomass was maximized when the long day length photoperiod was minimized (i.e., 14 days), while THC and CBD potency were maximized under long day length photoperiod for ~42 and 49–50 days, respectively (Table 1). This work reveals a yield trade-off in *C. sativa* between cannabinoid concentration and floral biomass where more time spent under long-day lighting maximizes cannabinoid content and less time spent under long-day lighting maximizes floral biomass (Figure 1). Growers should carefully consider the length of long-day lighting exposure as it can be used as a tool to maximize desired yield outcomes [52].

*C. sativa* has regained worldwide interest as a crop across temperate and subtropical regions. Nitrogen (N) is critical for maximizing yields in *C. sativa* [64], and its effects on plant physiology are broadly understood. *C. sativa* is a nitrophilic species and presents higher requirements of N. When *C. sativa* plants generate the most biomass during vegetative growth, N demand is higher than in the flowering stage [64]. Comparative study of growth responses of a temperate *C. sativa* variety (Morphet Late) and three tropical/subtropical varieties (ECO-GH15, ECO-MC16, and ECO-YP16) to tropical daylengths (11.5 h and 12.5 h), temperatures and varying nitrogen (0, 50, 100, and 150 kg ha^−1^ of N) rates revealed significantly different responses between varieties in terms of days to emergence, time to flowering, growth patterns, and final biomass showing predominant responses to daylength or temperature depending on the variety [64] (Table 1) However, more research is required to understand the plant responses when grown at those latitudes [64] (Table 1). *C. sativa* is a multi-purpose species and understanding the most suitable environment for each genotype depends on the final product required. This knowledge will be fundamental to improving productivity and helping emerging agricultural industries. These findings will help select varieties with particular physiological traits for new low latitude cropping tropical environments [64].

### 3.3. Effect of Light Intensity in Growth and Metabolism of C. sativa

Photosynthesis in plants is primarily driven by light within the 400–700 nm spectral range, known as photosynthetically active radiation (PAR). The efficiency of this process is largely dependent on photon irradiance in this range, measured as photosynthetic photon flux density (PPFD), expressed in μmol m^−2^ s^−1^. According to the International Commission on Illumination, light intensity is synonymous with photon flux density, which quantifies the number of photons hitting a surface per unit time and area. However, in the horticultural literature, PPFD (Figure 1) remains the standard term for photon irradiance within the PAR wavelength range [79].

Photosynthetic tissues experience a decline at light intensities (LI) below their light saturation points (LSP), where photosynthesis reaches its apex. However, for indoor-grown cannabis, whole-plant photosynthesis is optimized when the LI at the upper canopy leaves approaches their LSP. This optimization is facilitated by the attenuation of PAR within the canopy due to self-shading, allowing lower canopy foliage to operate within its optimal LI range. Unlike many other indoor crops, cannabis foliage exhibits an exceptional tolerance for exceedingly high LI, even when exposed to considerably higher PPFD levels than they are accustomed to. *Cannabis* leaves possess an extraordinary photosynthetic capacity, adeptly converting Photosynthetically active radiation (PAR) into biomass. This remarkable potential for biomass conversion underscores cannabis’s exceptional adaptability and resilience in high-light environments, rendering it an exceedingly efficient crop for indoor cultivation [79].

In a rigorous study, *Cannabis* plants were cultivated for 12 weeks under a strict 12 h light/12 h dark photoperiod with canopy-level PPFDs ranging from 120 to an impressive 1800 μmol m^−2^ s^−1^, using state-of-the-art LED technology. The research aimed to elucidate the intricate connections between light intensity (LI), photosynthesis, yield, and the overall quality of cannabis grown indoors. Findings revealed that leaf light response curves fluctuated with localized PPFD and throughout the flowering cycle, making them unreliable predictors of whole-plant yield responses to LI. Remarkably, dry inflorescence yield increased linearly with rising canopy-level PPFD up to 1800 μmol m^−2^ s^−1^, despite leaf-level photosynthesis saturating at much lower levels. Additionally, the density of the apical inflorescence and the harvest index surged with increasing LI, enhancing marketable tissue quality and significantly reducing waste. LI treatments did not affect cannabinoid potency and had only minor effects on terpene potency, indicating that increasing LI can substantially boost yields while maintaining consistent secondary metabolite profiles (Figure 1). Commercial growers can leverage these robust findings to determine the optimal LI for their production environments, balancing input costs against product value. Future research must expand to include multiple cultivars of both *indica*- and *sativa*-dominant biotypes and explore the synergistic effects of CO_2_ and LI on yield. A comprehensive cost-benefit analysis is essential to identify the optimal combination of these critical inputs, ensuring maximum efficiency and profitability in *Cannabis* cultivation [79].

A landmark study on industrial *C. sativa* assessed various agronomic traits, amino acids, saccharides, and their derivatives under four distinct light intensities: 30, 80, 130, and 180 μmol m^−2^ s^−1^ [47]. The findings were unequivocal: as light intensity increased, levels of amino acids and soluble proteins plummeted, whilst soluble sugars surged. The study pinpointed 130 μmol m^−2^ s^−1^ as the optimal light intensity, yielding the least stress for plant height, free amino acids, and soluble proteins [47]. Plant height diminished with higher light intensities, but stem diameter and biomass reached their zenith at 130 μmol m^−2^ s^−1^. This intensity also minimizes stress on amino acids and soluble protein content. For specific metabolites, 30 μmol m^−2^ s^−1^ proved optimal for amino acids and soluble proteins, whereas 180 μmol m^−2^ s^−1^ was superior for soluble sugars (Figure 1). Light intensity profoundly influenced crucial metabolites such as arginine, cysteine, isoleucine, leucine, methionine, phenylalanine, proline, maltose, fructose, glucose, and sucrose (Figure 1). The study conclusively demonstrated that industrial *C. sativa*’s morphological characteristics and metabolite profiles respond dramatically to varying light intensities. Achieving optimal results requires meticulously chosen light intensities tailored to specific objectives. These groundbreaking findings underscore that optimal light intensity can significantly enhance plant growth, amino acid content, soluble protein levels, and soluble sugar concentrations (Figure 1). This study sets a new standard for agronomic excellence, providing pivotal insights for future research and the cultivation of industrial *C. sativa* (Table 1) [47].

In the realm of indoor cannabis production, the propagation, vegetative growth, and flowering stages each demand meticulously tailored photoperiod and light intensity (LI) requirements [48]. Cannabis crops, depending on genotype, spend a critical 6 to 12 weeks under a stringent 12 h flowering photoperiod to reach peak maturity for harvesting. The vegetative stage, despite its brevity, imposes an intense energy demand due to significantly higher LIs and extended photoperiods (≥16 h) compared to the flowering stage. Achieving the optimal post-vegetative stage morphology is paramount and varies based on the cultivator’s production system [48] (Table 1). The overarching goal is to ensure exceptional transplant success and robust vegetative growth.

During the vegetative stage, LI plays a crucial role in shaping vital plant growth attributes such as height, stem thickness, branching, leaf size, leaf thickness, and biomass partitioning. These attributes are critical for the plant’s resilience and productivity as it transitions to flowering. Therefore, selecting the precise LI during this stage is essential to forge a formidable foundational structure, characterized by thicker stems and abundant nodes, which underpins prolific inflorescence development—accounting for more than half of the total aboveground biomass at peak maturity [48].

In a pivotal study exploring the impact of a wide range of LIs on vegetative-stage cannabis morphology and growth attributes under exclusive LED lighting, it was conclusively demonstrated that PPFD levels between 600 and 900 µmol·m^−2^·s^−1^ struck the perfect balance. This range optimized key morphological parameters while curbing energy consumption associated with excessively high LIs. These groundbreaking findings empower cultivators with the knowledge to fine-tune their LI strategies, harmonizing growth and morphological excellence with the economic imperatives of energy efficiency. While the ideal morphological and growth attributes of vegetative-stage cannabis plants are subjective to each genotype and production scenario, the identified LI response patterns offer cultivators a powerful tool to maximize their production goals [48] (Table 1). This optimization not only enhances growth and morphological characteristics but also judiciously balances the economic returns against the elevated input costs of providing more PAR to their crops [48]. This study sets a new benchmark for excellence in indoor cannabis cultivation, guiding the industry towards unparalleled efficiency and productivity.

Micropropagation revolutionizes the rapid and effective propagation and maintenance of uniform plantlets in sterile cultures, proving invaluable to high-value industries such as the burgeoning cannabis sector [51]. Yet, the microclimates within micropropagation vessels are plagued by low light intensity, limited CO_2_ availability, and excessive humidity, which severely hinder photo-autotrophy and induce stress-related ethylene accumulation. These adverse conditions complicate the development of robust organs, representing a significant drawback to in vitro plant maintenance. To combat these formidable challenges and bolster in vitro development, sugar is provided as a supplemental carbon source, facilitating photo mixotrophic metabolism [51] (Table 1).

However, to achieve superior plant quality and ex vitro success, cultivating photoautotrophic proficiency is paramount. The photoautotrophic potential of in vitro specimens is intricately affected by the interplay of light intensity, sugar concentration, and atmospheric factors [51]. Therefore, unraveling the complexities of in vitro plant physiology is of utmost importance. The dynamic balance of photoperiodic carbon gains and losses is a critical determinant of plant growth and development, with diurnal CO_2_ fluctuations being pivotal to biomass accumulation (Figure 1) [51]. Although CO_2_ generated during dark respiration is swiftly recaptured during light periods in vitro, managing gas dynamics within a closed system remains a daunting challenge. This issue is exacerbated by the presence of exogenous carbon sources such as sucrose, which can significantly alter metabolic processes. Resolving these challenges is essential for optimizing micropropagation conditions and ensuring the unparalleled success of in vitro plant cultivation in *C. sativa* [51].

The gas exchange system unveiled here represents a revolutionary leap in micropropagation, seamlessly integrating open flow/force ventilation, cutting-edge LED technology, and precise environmental control to scrutinize the effects of factors like CO_2_ concentration, sucrose, and light intensity on the photosynthetic prowess of cultured plantlets [51] (Figure 1). This state-of-the-art system, rigorously tested on the economically critical *C. sativa* L., addresses the paramount need for advanced micropropagation techniques. Traditional micropropagation methods often cripple photosynthetic performance, but this pioneering system shatters those limitations, opening new frontiers to dissect the intricate roles of light signaling and photosynthesis in mitigating in vitro morphophysiological disorders. By maintaining CO_2_ at meticulously controlled levels (400 and 1200 ppm) alongside finely tuned light intensities, we developed comprehensive photosynthetic light response curves, revealing the dynamic interplay of irradiance, CO_2_, and additional variables on photosynthetic efficiency. Furthermore, continuous monitoring of net carbon exchange rates (NCERs) over a 24 h light/dark cycle under standardized conditions provided precise estimates of relative growth rates (daily C-gain) (Table 1) [51]. This groundbreaking system equips researchers with an unparalleled tool to unravel the complexities of in vitro plant physiology and carbon dynamics, addressing questions previously deemed insurmountable [51]. This technological marvel sets a new benchmark in micropropagation, promising to elevate the cultivation of high-value crops like cannabis to unprecedented heights.

Although further experiments are essential to fully illuminate the intricacies of in vitro cannabis photosynthesis, these groundbreaking findings affirm the system’s unparalleled precision in measuring gas exchange related to photosynthetic capacity and carbon gain [51]. Leveraging advanced LED technology alongside meticulous gas exchange measurements catapults the speed, accuracy, and replicability of experimental procedures, propelling our understanding of photosynthesis to new heights. This cutting-edge system seamlessly integrates with machine learning, metabolomics, and genomics, enabling the precise modeling of the intricate interactions between light, CO_2_, and sucrose metabolism. Such integration optimizes plant development evaluations, refining our comprehension of co-active physiological processes [51] (Table 1). By meticulously modeling diverse physiological responses to critical environmental factors, this revolutionary approach promises to redefine plant tissue culture methods with unprecedented precision. The insights gleaned from this system will guide the evolution of in vitro micropropagation techniques and the strategic application of precision LEDs, elucidating the complex relationships between light intensity, quality, and sugar metabolism in plants—dimensions often neglected or challenging to conceive (Table 1). This innovative system stands as a beacon of progress, poised to revolutionize plant tissue culture and cultivation practices, enhancing precision and efficiency to an unprecedented degree [51].

*C. sativa* metabolites, particularly phenolic and terpene compounds like cannabidiol and cannabigerol (Figure 1), have immense medicinal value and potential for treating numerous incurable diseases. Light intensity is a critical factor influencing the synthesis of these potent plant metabolites. In a groundbreaking study on industrial *C. sativa* ‘Yunma 1’, researchers used advanced UPLC-MS/MS wide-target metabolomics technology to analyze the metabolic profile under various light intensities (Table 1) (30, 80, 130, and 180 μmol m^−2^ s^−1^) [49]. The findings were definitive: light intensity profoundly impacts the *C. sativa* metabolic profile. At 180 μmol m^−2^ s^−1^, levels of lipids, phenolic acids, flavonoids, amino acids and their derivatives, organic acids, alkaloids, nucleotides and their derivatives, sugars, alcohols, vitamins, cannabinoids, coumarins, lignans, and terpenoids were highest [49]. However, the synthesis of critical secondary metabolites, spirodienone, and iso-spirodienone (cannabispiradienone) peaked at 80 μmol m^−2^ s^−1^. Additionally, the concentrations of phenolic acids and flavonoids reached their maximum at 130 μmol m^−2^ s^−1^, corroborating previous research (Table 1) [49]. These results provide a crucial foundation for understanding the metabolic characteristics of specific secondary metabolites and identifying optimal conditions for their synthesis. This study underscores the paramount importance of light intensity in maximizing *C. sativa* metabolite production, setting a new standard for precision in agricultural practices [49].

### 3.4. Growth, Development, and Secondary Metabolism: Combined Effect of Light Spectrum, Photoperiod and Intensity on C. sativa

Research on light studies has primarily concentrated on assessing the isolated impacts of either spectral quality or photosynthetic photon flux density (PPFD) on inflorescence mass and concentrations of plant secondary metabolites (PSMs). However, there is an increasing awareness, evidenced in various plant species, that the interplay between spectral composition and PPFD significantly influences plant dry matter accumulation and PSM concentrations (Figure 1) [65]. In the context of medical cannabis cultivation, which utilizes diverse spectra and PPFD levels, the interactive effects of these factors on inflorescence mass and PSMs are garnering significant interest from both industrial stakeholders and the scientific community [65]. LED fixtures vary in their spectral output, from narrow to broad bandwidths, which can influence plant growth. Broadband spectra may provide more balanced light exposure and enhance plant dry matter production. Given the variability in plant responses to different spectra and PPFD, it is crucial to select appropriate lighting tailored to the needs of medical cannabis, though significant knowledge gaps remain in this area.

Recent investigations reveal a profound synergy between light spectrum and PPFD on dry matter production and inflorescence yield in medicinal cannabis [65]. White light with dual red peaks at 640 and 660 nm significantly outperforms white light with a single red peak at 660 nm, dramatically enhancing inflorescence yield and light use efficiency across all PPFD levels. This enhancement is attributed to superior total plant dry matter production and optimized plant architecture, maximizing photon capture [65]. Critically, the white light fraction and spectrum breadth exert no influence on inflorescence yield, with cannabinoid concentrations remaining consistent, ensuring premium PSM quality. At elevated PPFD, dual red peak light markedly increases terpenoid concentrations; at lower PPFD, photosynthetic efficiency improves, showing no effect at higher PPF (Table 1) [65]. Incorporating 640 nm alongside 660 nm markedly boosts light use efficiency and dry matter production [65]. As PPFD escalates, light energy seldom limits dry matter production, but pigment overexcitation can induce ROS formation, causing photo-oxidative damage toward photoinhibition (Figure 1). A higher white fraction, with a well-balanced red-to-blue ratio and enhanced green fraction, significantly mitigates photoinhibition risk, promoting superior quantum yields at high PPFD by ensuring balanced light absorption across pigments. This approach is pivotal for preventing photoinhibition and maximizing plant productivity [65].

The prevailing belief that increasing light intensity (LI) and ultraviolet radiation (UV) can significantly boost cannabis secondary metabolite levels lacks substantial scientific backing, leaving cultivators uncertain about lighting optimization [80]. A seminal study has now illuminated this area, revealing that cannabis plants subjected to an intense combination of UV and PPFD (via LED) demonstrated remarkable growth, with aboveground biomass metrics increasing by 1.3–1.5 times in the highest PPFD treatments compared to the lowest. The ‘Meridian’ cultivar’s inflorescence dry weight surged by 1.6 times. Moreover, plants in the highest PPFD treatment directed significantly more biomass to inflorescence tissues, resulting in a 7% increase in the harvest index (Table 1) [80]. Notably, the study detected no UV treatment effects on aboveground biomass metrics or inflorescence cannabinoid concentrations. However, sugar leaves exposed to UVA + UVB treatment exhibited a 30% increase in THC concentrations, although total THC in these foliar tissues remained unaffected by UV [80]. This study highlights the profound impact of UV and high PPFD on cannabis growth and secondary metabolite production, underscoring the critical need for the meticulous optimization of these protocols.

Designing an optimal UV exposure protocol for cannabis cultivation requires the meticulous consideration of several factors: spectrum, intensity, daily duration, and total exposure time relative to the harvest period. The scheduling of UV treatments within the PAR photoperiod is also crucial. Researchers must assess not only inflorescence yield and secondary metabolite composition but also the significant morphological impacts of UV on developing inflorescences and related tissues, such as the density and composition of glandular trichomes (Figure 1). *Cannabis* plants thrive under high canopy light intensities in indoor settings [80]. This study highlighted the economic benefits of maximizing canopy-level PPFD. However, it found no commercially significant advantages from UV radiation exposure [80]. Given the myriad potential UV exposure strategies, further research is essential to determine if UV can become a viable commercial tool in indoor cannabis production and to establish the most effective treatment protocols for commercial application.

Daylength-extension (DE) lighting is a vital technique in the cannabis industry, employed to enhance plant size and produce cuttings by extending the vegetative stage and controlling flowering [54]. Despite its importance, growers have encountered persistent issues with incomplete or transitional inflorescences in several cannabis cultivars, even under extended photoperiods. Notably, ‘Suver Haze’ in North Carolina nurseries exhibited incomplete inflorescences with a 15 h photoperiod [54]. A detailed investigation revealed that a 15 h photoperiod with DE lighting at 1 µmol m^−2^ s^−1^ PPFD kept ‘Suver Haze’ vegetative but still resulted in incomplete inflorescences (Table 1). In contrast, an 18 h photoperiod with the same DE light intensity successfully prevented flowering. Surprisingly, increasing DE light levels from 1 to 10 µmol m^−2^ s^−1^ PPFD did not resolve the issue of incomplete inflorescences [54]. This underscores the urgent need for comprehensive research to understand the effects of light pollution on flowering crops and identify the critical photoperiods required to prevent incomplete inflorescences in common cannabis cultivars [54]. Future studies must use the same genotypes or cultivars from consistent sources to ensure reliable and applicable results. The industry must address these challenges by leveraging rigorous scientific inquiry to refine DE lighting protocols and optimize cannabis cultivation outcomes.

## 4. Conclusions and Prospects

Light conditions, including intensity, spectrum, and duration, wield an extraordinary influence over morphogenetic responses, playing a pivotal role in regulating primary and secondary metabolism in plants such as *C. sativa*. Mastering metabolic adjustment is paramount for optimizing plant growth and development while minimizing the devastation of adverse environmental conditions. The intricate redox-mediated effects of light intensity, photoperiod, and spectrum on primary and secondary metabolism within individual cell compartments demand rigorous investigation, charting critical directions for future research. Light serves as a highly promising abiotic elicitor for stimulating the production of vital metabolites in various in vitro plant systems. Among the diverse abiotic elicitors, light has garnered significant attention due to its precise wavelengths, cost-effectiveness, and long-lasting nature, making it an ideal tool for enhancing secondary metabolite synthesis. Numerous studies have demonstrated that different light sources, with varying qualities and intensities, substantially boost secondary metabolite accumulation across multiple plant species under *in vitro* conditions. These findings open the door to a deeper exploration of light as a potent elicitor. However, the focus of most studies has been primarily on plant growth and development, as well as primary metabolite generation, underscoring the scarcity of comprehensive insights into the regulatory factors governing light-induced elicitation mechanisms. As a result, many investigations fail to fully elucidate the mechanisms by which light enhances the production of pharmacologically valuable secondary metabolites, as these mechanisms likely vary depending on plant species, culture conditions, and the specific light source applied.

As our comprehension of hormone pathways and photomorphogenic processes deepens, it becomes imperative to unravel the mechanisms through which light signaling pathways intersect with hormone signaling in *C. sativa*. Groundbreaking advances in light-regulated hormone signaling technologies now empower researchers to delve into the spatiotemporal patterns of signaling as perceived by receptors. The burgeoning understanding of transcriptional and protein regulation presents formidable challenges but is essential for pioneering future progress. Artificial lighting in horticulture has long been a cornerstone for assimilation and photoperiodic functions. However, recent monumental advancements in plant photomorphogenesis and metabolism have heralded the era of state-of-the-art lighting systems and innovative strategies, such as photo-selective greenhouse covers, to manipulate light for supreme control over plant development and metabolism. This revolutionary approach promises to redefine and elevate the optimization of plant growth and productivity through masterful light manipulation. More species and variety-specific trials and research can elucidate the undiscovered molecular mechanism underlying the physiology and metabolism of *C. sativa*.

## Figures and Tables

**Table 1:** Effects of light spectrum, intensity, and photoperiod on growth and metabolic changes in various cannabis varieties.

Sl. No.	Variety	Effect of Light (Spectrum, Intensity, Photoperiod)	Duration	Growth Condition	Growth and Developmental Changes	Metabolic Changes	References
**1.**	WR, CCBD	Red light (600–700 nm), blue light (400–500 nm), far-red light (700–800 nm)	21 days	Temperature: 25–28 °C RH: 65–85%.	Red and blue light did not significantly affect rooting. Far-red light improved adventitious rooting and stem elongation.	Blue and red lights were not highlighted in this research. Far-red light may induce auxin biosynthesis and carbohydrate content in stem cuttings.	[46]
**2.**	Yunma 1	Different light intensities using LED (30 μmol m^−2^ s^−1^, 80 μmol m^−2^ s^−1^, 130 μmol m^−2^ s^−1^, 180 μmol m^−2^ s^−1^)	30 days	Temperature: 24/22 °C day/night RH: 60% Photoperiod: 16 h light/8 h dark	Stem diameter and root dry and fresh weight increased at 130 μmol m^−2^ s^−1^. Decreased plant height with increasing light intensity (180 μmol m^−2^ s^−1^).	Amino acids, soluble proteins content decreased with increasing LI. Sugar content increased with increasing LI.	[47]
**3.**	Gelato	Five light-intensity target levels (200, 450, 700, 950, and 1200 μmol m^−2^ s^−1^)	21 days	Daytime temperature and RH: 26 ± 3 °C and 30 ± 9%, 26 ± 3 °C and 30 ± 8%, and 25 ± 2 °C and 30 ± 8%. Nighttime temperature and RH: 23 ± 2 °C and 37 ± 5%, 23 ± 2 °C and 36 ± 5%, and 23 ± 2 °C and 36 ± 5%	Plant height, growth index, nodes, stem thickness, and aboveground dry weight grew quadratically and asymptotically with light LI. PPFD levels between 600 and 900 μmol m^−2^ s^−1^ appeared to achieve an appropriate balance in optimizing key morphological parameters.	This experiment found no influence on blue or red light. Photosynthetic capability increased with light intensity.	[48]
**4.**	Yunma 1	Different light intensities (30 μmol m^−2^ s^−1^, 80 μmol m^−2^ s^−1^, 130 μmol m^−2^ s^−1^, and 180 μmol m^−2^ s^−1^ using LED (red, blue and white)	30 days	Temperature: 24 °C/22 °C (day/night) RH: 60% Photoperiod: 16 h/8 h	Significant impact on plant development, photosynthesis, and antioxidant enzyme activity in different light intensities.	High light intensity increased *C. sativa* metabolic profile, lipids, phenolic acids, flavonoids, amino acids, organic acids, alkaloids, nucleotides, sugars, alcohols, vitamins, cannabinoids, coumarins, lignans, terpenoids, tannins, and carbohydrates.	[49]
**5.**	FINOLA	Red 600–700 nm, far-red 700–800 nm, short wavelength (blue 400–500 nm, UV-A 315–400 nm, and UV-B280–315 nm) radiation	78 days	Temperature: 24/22 °C day/night RH: 60%/50% day/night	Low R/FR increased plant height and reduced inflorescence production. Short-wavelength radiation did not affect plant morphology or inflorescence yield.	Compared to low R/FR ratio treatment, high R/FR ratio treatment enhanced CBD, THCVA, CBGA, and terpene concentrations. Monoterpene and myrcene concentrations rose with UV-B. No individual light effects were discussed.	[50]
**6.**	Cultures of *C. sativa*, accession RTG-XX	LEDs emitted blue (456 nm), red (657 nm), and some white light	--	Temperature: 27 °C Photoperiod: 18 h, PPFD: 50 μmol m^−2^ s^−1^	Long-term in vitro photosynthesis and respiration in response to various growth conditions.	--	[51]
**7.**	*Cannabis sativa*	Photoperiod	50 days	Long-day (≥18 h of light) and short-day lighting (≤12 h of light)		Morphological characteristics were enhanced.	[52]
**8.**	Bamahuoma	White light (control) (WL), blue light (BL) (450 nm), red light (RL) (650 nm), and 50% blue light with 50% red light (RBL)	14 days	Temperature: 25/20 °C (day/night), RH: 70–90%	BL boosted the shoot’s fresh and dry biomass, number of leaves/plants, stem diameter, root length, and chlorophyll concentration compared with WL.	BL increases net photosynthesis, stomatal conductance, and transpiration, and decreases lipid peroxidation, superoxide dismutase, and peroxidase activity. RL and RBL significantly reduced the plant biomass and gas exchange parameters with enhanced antioxidant enzyme activities.	[40]
**9.**	Meridian	PPFD of either 600, 800, or 1000 μmol m^−2^ s^−1^ for 12 h day^−1^ or PPFD of 600 μmol m^−2^ s^−1^ plus ultraviolet (UV, 280–400 nm) for either 12 h day^−1^ of 50 μmol m^−2^ s^−1^ from LEDs with a peak wavelength of 385 nm for 45 days (UVA) or 5 h day^−1^ of 3 μmol m^−2^ s^−1^ of wideband ultraviolet fluorescent lighting (UVA + UVB).	45 days	Temperature and RH: 26 ± 1.2 °C and 40 ± 6.9% (day) Temperature and RH: 22 ± 1.9 °C and 47 ± 3.9% (night)	High LI can substantially increase cannabis yield compared to UV light. Above-ground biomass metrics were 1.3–1.5 times higher in the highest vs. lowest PPFD treatments.	Total foliar THC concentration was unaffected by UV exposure. Sugar leaves (i.e., small leaves associated with inflorescences) of plants in the UVA + UVB treatment had ≈30% higher THC concentrations. High PPFD levels can substantially increase cannabis yield	[53]
**10.**	Stock plants of ‘Suver Haze’	Day-length extension lighting with four light intensity treatments of 1.0, 2.5, 5.0, and 10.0 μmol m^−2^ s^−1^ PPFD	24 days	Temperature: 26 °C RH: 85% to 95% Photoperiod: 16 h PPFD: 120 μmol m^−2^ s^−1^	The DE impacts stem length, nodes, and roots. The photoperiod and light intensity affect blooming and inflorescence.	---	[54]
**11.**	‘Gelato-27’, ‘Grace’, and ‘Meridian’	Blue, red, UV-A, white, and fluorescent LEDs. The photon flux ratios of blue (B; 400–500 nm) and red (R; 600–700 nm) narrowband LED treatment combinations were (1) BR, fixed spectrum of B15/R85; (2) B, B75/R25 on day 0–2 followed by B15/R85 on day 2–14; (3) B+UVA, B75/R25 on day 0–2 followed by B15/R85 on day 2–14 plus 15 μmol m^−2^ s^−1^ of UVA on day 7–14; (4) B50, B15/R85 on day 0–7 followed by B50/R50 on day 7–14	21 days	Temperature and RH: 26 ± 3 °C; 30% ± 9%, 26 ± 3 °C; 30% ± 8%; 25 ± 2 °C, and 30% ± 8% (day) Temperature and RH: 23 ± 2 °C; 37% ± 5%, 23 ± 2 °C; 36% ± 5%; 23 ± 2 °C, and 36% ± 5%	Blue light increased stem thickness and root index compared to other treatments. No spectrum treatment effects on the percentage of cuttings that rooted, and root index values were higher in cuttings grown under B+UVA vs. F. Relative root dry weights of plugs from the B, B+UVA, B50, and F treatments were higher than the W treatments. Spectrum treatments did not affect the chlorophyll content index. Cuttings under B had thicker stems than BR and W, and those under F showed the lowest percentage of new above-ground growth.	---	[55]
**12.**	Finola and USO31	Plants were grown under white (W) and purple (P) light at different photoperiods (16/8, 20/4, and 24/0)	35 days	LED light (~80 μmol m^−2^ s^−1^, 22 ± 2 °C)	White light yielded the highest PI_total_. Purple light yielded the lowest PI_total_.	A 16/8 photoperiod, regardless of the light type, yielded the lowest TBARS contents.	[56]
**13.**	Kanada and E19	Red and far-red ratio (R/FR) from ceramic metal–halide lamps (CMHs) and high-pressure sodium lamps (HPSs), LEDs	84 days	Night temperature: 18 °C Day temperature: 23.5°C Humidity: 80%	Modulate the photosynthetic rate	Light spectra significantly influenced CBDA and terpene concentrations	[57]
**14.**	‘Babbas Erkle Cookies’ accession	Blue light (430 nm), red light (630 nm), rose light (430 + 630 nm, ratio 1:10), purple light (430 + 630 nm, ratio 2:1), and amber light (595 nm)	70 days	Temperature: ~28 °C RH: 40–55% (day) Temperature: 25–27 °C, RH: 50–65% (night) Photoperiod: 18 h light (vegetative), 12 h light (flowering)	LED light treatments had lower fresh mean inflorescence mass than the control (HPS, 133.59 g plant^−1^), and monochromatic blue light yielded the least fresh inflorescence mass (76.39 g plant^−1^)	Blue light increased the concentration of cannabinoids, including THC and CBG, as well as terpenes. Blue light had a lesser impact on cannabidiol (CBD) biosynthesis.	[58]
**15.**	Cannatonic, Hindu Kush, and Northern Lights	10/14 h (light/dark), 12/12 h (light/dark), 14/10 h (light/dark)	75 days	Temperature: 25 °C RH: 50%	Flower biomass yields were highest for all lines when treatments started with 14L/10D.	A 14L/10D photoperiod significantly decreased THC concentration. Conversely, in Cannatonic, all 14L/10D treatments significantly increased CBD concentration.	[59]
**16.**	Black Triangle, Garlic Jelly, Ghost Train Haze, Powdered Donuts, Chem de la Chem, Legendary Larry, Gorilla Glue, OG Kush, Incredible Milk, Blue Dream	All treatments were based on a standard 24 h day and included 12 h, 12.5 h, 13 h, 13.5 h, 14 h, and 15 h of light. Approximate light intensity of 360 μmol m^−2^ s^−1^ from white LEDs	3–4 weeks	Temperature: 25.00 ± 0.84 °C RH: 78.40 ± 8.80%	Flowering initiation occurred in all cultivars under all photoperiod treatments up to 14 h. Delays in flowering initiation between 14 h and 12 h varied from 0 to about 4 days, depending on the cultivar. Some cultivars began flowering under 15 h conditions, but floral tissues did not progress beyond the initiation phase.	---	[60]
**17.**	---	Red light (600–700 nm), Green light (500–600 nm), Blue light (400–500 nm)	14 weeks	Day temperature: 23.2 °C ± 0.1 °C RH: 40.7% ± 0.1% Night temperature: 22.2 °C ± 0.1 °C RH: 41.1% ± 0.3%	Blue light resulted in a higher bud yield than white light.	THC/CBD was unaffected by light. In individual light effects, no phytochemical alterations were identified.	[44]
**18.**	Finola	Blue and white light	30 days	Temperature: 20 °C Photoperiod: 16/8 h (light/dark)	White light increased plant shoot and root length.	Leaf pigments were higher under blue light. DPPH, FRAP, flavonoids, and total flavanol content; phenolic acids were not influenced by light type.	[61]
**19.**	High THCA variety, balanced CBDA/T CA variety, high CBDA variety	White LED, two ratios of blue + red LED (1:4 and 1:1), and a spectrum generated by HPS	58 days	Temperature: 25 °C, RH: 50%	The highest inflorescence yields occurred when the spectrum was limited to a 1:1 red-to-blue ratio, and in two of the three varieties tested, a 1:4 blue-to-red ratio produced similar results. The lowest yields were observed under white light with a 1:1 blue-to-red ratio.	Blue-rich light-enhanced CBGA accumulation more than far-red-rich HPS light.	[15]
**20.**	Xinma	CK, high-pressure sodium as light source, (R/B (ratio of red light to blue light) 9.30:1, PPFD (photosynthetic photon flux density) 19:1); LED1 (R/B 9.20:1; PPFD 129); LED2 (R/B 1.61:1; PPFD 540); LED3 (R/B 6.47:1; PPFD 28.2); LED4 (R/B 7.15:1; PPFD 41.7); LED5 (R/B 16.8:1; PPFD 252)	110 days	Temperature: 24 °C Photoperiod: 16/8 h (day/night)	LED2 and LED5 effectively sustained *C. sativa* growth in plant height, stem diameter, and leaf numbers compared to CK. LED1, LED4, and LED3 treatments, which significantly reduced aboveground biomass, whereas LED2 and LED5 enhanced it. Flower biomass for LED2 and LED5 significantly increased compared to CK, while other LED treatments notably decreased flowers yield.	LED2 could significantly increase the CBD content of both leaves and flowers compared to CK. LED5 only notably increased the CBD content of leaves among the LED treatments.	[8]
**21.**	*C. sativa* L. strain India	Green, far-red, and UV-A	25 days	Temperature: 23–27 °C Photoperiod: 16 h PPFD: 300 μmol m^−2^ s^−1^	--	THC levels were higher in plants exposed to white, R8/B2, and R7/B2/G1 light. THCA accumulation was elevated in R6/B2/G1/FR1 and R5/B2/W2/FR1 light treatments, which coincided with reduced photosynthetic rate and increased ROS, total phenols, total flavonoids, DPPH radical scavenging capacity, and antioxidant enzymatic activities. The R6/B2/G1/FR1 light treatment resulted in higher CBDA accumulation, increased stress-modulated substances, and reduced physiological traits.	[62]
**22.**	Stillwater cultivar	PPFDs ranging from 120 to 1800 μmol m^−2^ s^−1^	84 days	Day temperature: 25.3 ± 0.4 °C RH: 60.5 ± 4.8% Night temperature: 25.2 ± 0.3 °C RH: 53.1 ± 3.3%	Dry inflorescence yield exhibited a linear increase with increasing canopy-level PPFD up to 1800 μmol m^−2^ s^−1^, whereas leaf-level photosynthesis saturated well below 1800 μmol m^−2^ s^−1^.	There were minor LI treatment effects on the potency of cannabinoids and terpenes. The individual light effect is not specified.	[63]
**23.**	*C. sativa* L. strain India	L1, natural light. L2, W; L3, R8/B2; L4, R7/B2/G1; L5, R7/B2/FR1; L6, R6/B2/G1/FR1; L7, R5/B2/W2/FR1; L8, R5/B2/G1/FR1/UV1; L9, R6/B2/FR1/UV1; L10, R4/B2/W2/FR1/UV1; L11, R2/B2/G2/W2/FR1/UV1	20 days	Temperature: 30 °C (day), 25 °C (night) RH: 60–70% Photoperiod: 12 h light	L10 and L11 exhibited more leaves and nodes, while L2, L3, and L5 showed increased leaf length and leaf width. Higher shoot lengths were noted in L3, L6, and L9.	L3 treatment exhibited higher levels of chlorophyll a, b, and photosynthetic quantum yield (Fv/Fm). L4, L6, L8, and L11 demonstrated a higher node count with elevated osmolyte content, including proline, ascorbic acid, total soluble carbohydrate, and sucrose.	[37]
**24.**	Morphet Late, ECO-YP16, ECO-GH15, and ECO-MC16	600 W high-pressure sodium and eight 40 W globes suspended over the plants. Light intensity at canopy level (40 cm from the light source) was 300–350 μmol m^−2^ s^−1^ PAR	---	**Env.1:** Daylength: 12.5 h Temperature: 27 °C/24 °C night (day/night) **Env.2:** Daylength: 11.5 h Temperature: 27 °C/24 °C night (day/night) **Env.3:** Daylength: 11.5 h Temperature: 22 °C/10 °C (day/night)	Tropical daylengths, temperatures, and nitrogen levels impacted *C. sativa* growth parameters. They play a crucial role in regulating flowering initiation, flower development, stalk diameter, and above-ground biomass in *C. sativa* varieties.	Environmental factors such as day length and temperature significantly influence cannabinoid concentrations. Selecting low-THC varieties was emphasized for production in tropical/subtropical environments.	[64]
**25.**	King Harmony (Chemotype II, 1:1.5THC: CBD)	LED light, two low-white spectra (7B-20G-73R/narrow and 6B-19G-75R/2 peaks), and two high-white (15B-42G-43R/narrow and 17B-40G-43R/broad) spectra (600 and 1200 μmol m^−2^ s^−1^)	56 days	**Long-day phase:** 28/24 °C, 27/22 °C, 26/22 °C, 25/22 °C **Short-day phase:** Days 0–28; 28/24 °C, Days 29–42; 27/22 °C, Days 49–56; 26/22 °C	The 6B/19G/75R (dual 640 and 660 nm peaks) enhanced inflorescence weight through increased dry matter production, while 7B-20G-73R (single 660 nm peak) reduced it. Two high-white spectra (15B-42G-43R/narrow and 17B-40G-43R/broad) maintained inflorescence weight and increased dry matter production at high PPFD.	The 6B-19G-75R (dual peaks: 640 and 660 nm) spectrum elicited an increase in terpenoid concentrations at high PPFD, whereas other light spectra did not induce significant alterations in cannabinoid levels.	[65]

**Abbreviations:** LED—light-emitting diodes; LI—light intensity; R/FR—red/far-red; CBD—cannabidiol; THC—tetrahydrocannabinol; THCVA—tetrahydrocannabivarinic acid; CBGA—cannabigerolic acid; UV-B—ultraviolet-B; PFD—photon flux density; DE—daylength extension light; Fv/Fm—Fv/Fm is a normalized ratio created by dividing variable fluorescence by maximum fluorescence; RH—relative humidity; DPPH—2,2-diphenyl-1-picrylhydrazyl; FRAP—fluorescence recovery after photobleaching; PPFD—photosynthetic photon flux density.

## Data Availability

Data will be available upon request.
